# Supplementary material for: Are carbon emissions trading and green financial instruments synergistic? -Comprehensive quantitative research based on content analysis
Source: PLoS One. 2024 Mar 7;19(3):e0298601. doi: 10.1371/journal.pone.0298601 (PMC10919601; doi:10.1371/journal.pone.0298601)
Supplement: S1 Data — (ZIP) [file pone.0298601.s001.zip › Data/地级市Z_F工作报告.zip!/╣π╢1⁄2/╒╪╟∞╩╨/└┤╫╘╣1⁄2╓┌║┼í╛┬φ┐╦╩2╛▌═°í┐_2012.docx]

各位代表：

　　我代表肇庆市人民政府向大会作工作报告，请予审议，并请政协委员和其他列席人员提出意见。

　　 本届政府工作回顾

　　2011年是本届政府任期最后一年，也是“十二五”发展开局之年。一年来，面对后金融危机时期复杂多变的国内外环境，在省委、省政府和市委的正确领导下，在市人大及其常委会和市政协的监督支持下，市政府深入贯彻落实科学发展观，按照把肇庆建设成为能够代表珠三角科学发展成果城市的新要求，全面实施珠三角《规划纲要》，加快转型升级，建设幸福肇庆，推动全市经济社会持续快速健康发展，圆满完成市十一届人大六次会议确定的目标任务，实现“十二五”发展开门红。

　　——经济发展上新台阶。预计本地生产总值1330亿元，比上年增长（以下简称增长）15%，超过预期目标；地方财政一般预算收入92.23亿元，增长25.56%；规模以上工业增加值520亿元，增长27.8%；农林牧渔业增加值223亿元，增长6.5%；社会消费品零售总额396亿元，增长19%；主要旅游景区（点）接待游客2438万人次，旅游收入142.3亿元，分别增长21.4%、38.5%；外贸进出口总额53.4亿美元，增长21.6%，出口增幅居全省前列；商品房销售面积、销售额分别增长17%和38%。城镇居民人均可支配收入19020元，增长13%；农村居民人均纯收入8728元，增长16%。

　　——转型升级效果明显。编制完成市主体功能区规划和产业发展规划，获得珠三角地区转变经济发展方式创新工作综合三等奖。申报省级以上科技项目91项，35家企业与高等院校和科研院所签订产学研合作项目，肇庆新型电子元器件产业成为省市共建战略性新兴产业基地，中巴软件园被认定为首批省级现代服务业集聚区和省“两化”融合创新中心。新认定高新技术企业19家，新建省级企业重点实验室和工程技术研发中心各1家，鸿特精密在创业板上市，风华高科等7家企业被认定为省战略性新兴产业骨干企业和培育企业，星湖科技被评为省自主创新标杆企业。肇庆市和端州、鼎湖、高要获评全国科技进步先进市（区）。

　　——区域合作不断深化。协同广佛制定出台《广佛肇经济圈发展规划（2010－2020）》，确定第二批46个重点合作项目，广佛肇（怀集）经济合作区有序推进。“五个一体化”规划深入实施，“十大工程”项目建设进展顺利，广佛肇城际轨道建设扎实推进，完成广佛肇高速一期工程勘察设计招标，肇花高速加快筹建。粤桂产业合作示范区已列入《“十二五”粤桂战略合作框架协议》，我市与梧州市签署相关合作协议。

　　——招商引资成效显著。积极参加对央企、世界500强、民企500强和波兰、南非招商活动，成功举办央企战略合作座谈会和赴日本、新加坡等招商活动。第九届“肇庆金秋”签约、投产、动工项目124宗，投资总额971.18亿元。全年实际吸收外资10.2亿美元，增长9%；引进超千万美元项目73宗，投资总额17.8亿美元。承接产业转移项目150个，投资总额260亿元。

　　——重大项目加快推进。完成固定资产投资763亿元，增长22%。142项重点建设项目完成投资269.6亿元。华浩光电等13个重点项目建成投产，国电（大旺）热电联产、中恒医药、大唐热电等项目进展顺利。二广高速怀集至连州段和至贺州段、江肇高速二期加快建设，贵广、南广铁路肇庆段工程稳步推进。景丰联围加固达标工程基本完成。

　　——县域经济实力增强。各县（市）充分发挥资源环境优势，加快发展特色产业，推动县域经济快速发展。四会积极引进世界500强和央企投资项目，经济综合发展力居全省县域地区第三名。高要以倒逼机制推进转型升级，生产总值和财政一般预算收入居全市首位。广宁三大产业基地发展初具规模，特色经济集聚效应逐步显现。德庆产业转移工业园获评省级优秀园区，竞标成为省首批水利建设示范县并获得4亿元省级水利资金。封开华润水泥、圆珠顶铜钼矿等大项目加快推进，实际利用外资居全市山区县首位。怀集资源型特色产业发展加快，新城区开发加快推进。

　　——城乡建设展现新貌。启动肇庆新区规划论证工作。大力推进“城市建设管理年”活动，星湖牌坊公园、砚都大道、文明北路、肇庆大道、西江路等建设改造工程基本完成，首批天然气公交车和一批“百姓路”、“百姓灯”投入使用，完成市垃圾场扩容一期工程和78座病险水库除险加固任务。“三旧”改造动工项目49个，面积3050亩，端州龟顶新城初具规模。我市再次被确认为国家卫生城市，获评中国十佳宜游城市。加强生态建设保护，在全省率先启动生态景观林带工程建设，全市造林26.17万亩，绿化自然村160个，新建生态文明村314个。

　　——社会事业蓬勃发展。全市一半以上乡镇创建成为省教育强镇，端州、鼎湖、四会、高要通过省教育强区（市）督导验收，端州城区义务教育规范化学校覆盖率达95%以上。市第一人民医院新院主体工程、新医改五项重点工作基本完成。市图书馆新馆加快建设，端砚文化村被列为全国文化产业示范基地，端砚文化特色产业集聚园和古宋城保护与开发利用项目被列为“省新十项工程”。成功举办市第九届运动会。城镇基本养老、基本医疗、失业、工伤和生育保险参保人数均超额完成任务。完成104所乡镇敬老院改造扩建，8.6万名困难群众纳入低保范围。省委、省政府提出的扶贫开发“双到”工作三年任务，我市两年基本完成，走在全省前列。综治维稳更加扎实，公众安全感不断提升。劳动关系更加和谐，高新区被评为全国模范劳动关系和谐工业园区。

　　——十件实事扎实有效。向全市低收入群众发放临时性价格补贴2223万元。就业培训4.25万人，新增就业5.12万人，均超额完成任务。建成“四大蔬菜基地”共8347亩，挂牌营业“平价商店”66家。新增保障性住房6549套，超额完成省下达任务，农村低收入住房困难户住房改造完成8551户。新增优质幼儿园33所，市一中新校区动工兴建。阅江大桥正式动工。端州路人行天桥投入使用，过街隧道加快建设。环星湖绿道全线贯通，绿道管理维护不断完善。鼎湖山新入口广场完成规划设计等前期工作。城区出入口美化、亮化工程成效明显，主干道管理进一步加强。

　　——行政效能不断提升。开展创建“人民群众满意服务窗口”活动，据国家统计局肇庆调查队调查结果显示，群众对服务窗口总体满意率达89%。进一步简政放权，完成市第五轮行政审批制度改革，行政审批事项大幅减少。市行政服务中心服务事项增加到300多项，有效提高了办事效率。

　　各位代表，2011年各项工作任务的顺利完成，标志着本届政府各项目标任务圆满完成。五年来，我们坚持发展第一要务，狠抓工业主战场，加快招商引资和重大项目谋划建设，经济综合实力不断增强；我们坚持抢抓发展机遇，大力实施珠三角《规划纲要》，一体化发展步伐加快；我们坚持城乡协调发展，着力破解发展不平衡难题，区域竞争力明显提升；我们坚持以人为本，大力发展以民生为重点的社会事业，人民生活水平不断提高，在科学发展道路上迈出了更加坚实的步伐。

　　过去五年是我市经济发展最快的五年。预计实现GDP翻番，年均增长16%。地方财政一般预算收入年均增长29.85%。规模以上工业总产值超2000亿元，增加值增长3.2倍，年均增长33.4%，规模以上工业企业由788家增加到1035家，民营经济发展壮大，成为经济快速发展重要支柱。实施重点项目277个，总投资862.5亿元，累计完成固定资产投资2495亿元，年均增长28.6%。引进建设投资10亿元以上项目56个，五年共吸收外资44.7亿美元。社会消费品零售总额和进出口总额分别增长136%和94.6%。

　　过去五年是城乡面貌变化最大的五年。交通基础设施日趋完善，累计完成交通投资325亿元，新建及在建高速公路240.5公里，321、324国道和一批省、县道改造全面完成，采取政府回购等方式撤销肇庆大桥等10个收费站点，肇庆新港建成营运，西江航道肇庆段整治基本完成，高速铁路、城际轨道加快建设。城镇化水平不断提高，编制实施创建宜居城乡规划和土地利用总体规划，完成端州城区一批市政重点工程建设，城东新区初具规模。加快培育特色旅游，获 “广东省旅游综合改革示范市”、“国民旅游休闲示范市”称号。生态环保建设进一步加强，投资40多亿元建成水利工程1300多宗，水安全保障和水资源支撑能力全面提升。治理地质灾害成效明显，完成农田基本改造整治30万亩。通过“十一五”期间全市耕地保护目标责任制考核。新建19座城镇污水处理厂和配套污水管网，实现“一县一厂”目标，中心区8个中心镇建成污水处理厂，全市城镇生活污水处理率从33%提高到70.5%，污染减排任务全面完成，连续两年被评为省节能先进地区。建成区新增绿地面积900多公顷，修建绿道810公里，全市森林覆盖率达67.7%，成为省林业生态市。创建省卫生镇21个、省卫生村2095个、生态文明村2316个，均居全省前列。

　　过去五年是人民群众受益最多的五年。城镇居民人均可支配收入、农村居民人均纯收入分别增长64.7%和82.7%。“五大民生工程”和为民惠民实事扎实有效，九类民生支出从27.85亿元增加到70 多亿元，占财政一般预算支出60%以上。社会保障体系不断完善，实现城镇基本养老、基本医疗、失业、工伤和生育保险五大险种市级统筹，被征地农民养老保障实现即征即保，新型农村和城镇居民社会养老保险覆盖面不断扩大，企业退休人员人均养老金年均增长13.8%。累计发放城乡低保金3.02亿元，五保老人集中供养率提高到30%以上，社会救助体系逐步完善。累计新增城镇就业22.9万人，城镇登记失业率控制在3%以内。新建、改建一批中小学和幼儿园，新增优质学位近20万个，提前5年实现普及从学前到高中阶段15年教育目标，建成全省首个市级职教公共实训中心，职业教育办学规模居全省前列，高等院校教学质量不断提升。扎实推进文化强市建设，文化精品不断涌现，文化产业加快发展。公共卫生服务体系逐步完善，重大传染病得到有效防控。全民健身活动蓬勃开展，竞技体育再创佳绩。

　　过去五年是应对困难挑战最突出的五年。面对国际金融危机的冲击，我们全面贯彻“三促进一保持”方针，果断出台减免缓收部分行政性收费等政策措施，全力保增长、促发展。面对2008年低温雨雪冰冻以及每年旱涝台风等自然灾害，我们全力救灾复产，累计投入灾后重建复产资金4亿多元，保障人民群众生命财产安全，确保农业生产稳定。面对四川汶川特大地震灾害，我们广泛发动社会捐赠，积极承担克枯乡灾后重建任务，累计投入1.37亿元，提前一年超额完成援建任务。面对近两年国家宏观调控政策变化和资源环境约束趋紧的新形势，我们积极帮助企业解决融资、用地、用电、用工等困难，扶持企业转型升级，努力促进经济平稳较快发展。

　　过去五年是发展亮点最显著的五年。肇庆从国家层面整体纳入珠三角一体化发展，广佛肇经济圈建设加快推进。实现 GDP超千亿，财政一般预算收入增长2.7倍。建成3个省级产业转移工业园、2个省级产业集群升级示范区，肇庆高新区升级为国家级高新区。二广高速怀集至三水段建成通车，结束了我市山区县没有高速公路的历史。各县（市、区）发挥优势，提速发展。端州引进建设龟顶新城、华南智慧城等重大项目，现代服务业加快发展。鼎湖着力引进广新集团、中储粮物流等一批重大项目落户，发展后劲增强。四会加快建设肇庆东大门，推动产业集聚和企业增资扩产成效突出。高要狠抓招商引资和城市建设，成为全省首个地方财政一般预算收入突破10亿元的山区县（市）。广宁“辟山建园、再造一城”，林浆纸一体化、环保建材等支柱产业加快发展。德庆积极推进工业产业集聚发展，新农村建设走在全省前列。封开以华润水泥为龙头，全力打造省新型建材基地。怀集依托矿产、林木、水电等资源优势，加快培育壮大资源型支柱产业。

　　五年来，我们大力推动改革创新，不断增强发展活力。市、县两级政府机构改革和县级大部制改革试点顺利完成，简政强镇事权改革、事业单位分类改革和人事制度改革有序开展，实行三轮行政审批制度改革。公共财政体制框架基本建立。市属国有企业重组整合加快，3家骨干企业成功引入战略投资者。创新金融服务机制，端州农村商业银行、德庆华润村镇银行挂牌营业，金融机构存、贷款余额持续增加。农村综合改革力度加大，完成集体林权制度改革。旅游体制改革取得新进展。医药卫生体制改革和文化体制改革扎实推进。加快人才引进和科技创新步伐，深化校市和产学研合作，共获得省级以上科技计划项目立项350项。

　　五年来，我们大力维护社会和谐稳定，不断探索社会管理创新。开展“平安创建”活动，荣获全国综治“长安杯”。建立市、县两级政府应急管理机构，实行政府领导定期接访制度，有效预防和妥善处置群体性事件。安全生产和粮食安全责任制有效落实，打私、打假、打传和产品质量、食品药品安全、市场价格监管不断强化。完成省下达人口计划任务。国防后备力量建设不断推进，连续六届被评为省双拥模范城。统计、外事、侨务、港澳、民族、宗教、老区建设、人防、地震、气象、档案、地方志、妇女儿童、老龄、残疾人、慈善等工作不断取得新成效。

　　五年来，我们大力打造为民务实廉洁高效政府，不断加强自身建设。认真执行市人大及其常委会各项决议并向其报告工作，坚持向市政协通报情况，自觉接受监督。按期办结人大代表建议469件和政协提案755件。认真听取各民主党派、工商联、无党派人士、各人民团体和专家顾问意见建议。深入推进依法治市工作，圆满完成“五五”普法任务，启动“六五”普法教育。坚持依法民主决策，落实社会公示和听证制度。加强行政执法监督和行政复议。建立完善督查制度，加强效能监察，实施行政问责制和责任倒逼机制。全面推行政务、厂务和村务公开，开通“行风热线”和网络问政等新平台。严格执行党风廉政建设责任制，在全国率先创建党风廉政信息公开平台，加强行政监察、审计监督，治理商业贿赂、查处违法违纪案件、纠正损害群众利益不正之风成效明显。

　　以上成绩的取得，是省委、省政府和市委正确领导以及市人大、市政协监督支持的结果，凝聚着全市人民的智慧和汗水，离不开社会各界大力支持。在此，我代表市人民政府，向全市工人、农民、干部职工、外来务工人员、驻肇单位、驻肇部队、武警官兵以及社会各界人士致以崇高的敬意！向关心支持我市建设发展的外来投资者、港澳台同胞、海外侨胞和国际友人表示衷心的感谢！

　　我们也清醒地认识到，我市经济社会发展还存在不少困难和问题：经济欠发达、城乡区域发展不平衡问题仍然突出；产业集聚度不高，自主创新能力不强，产业转型升级有待加快；企业综合成本上升，用地、用电、融资、节能减排等压力较大；基本公共服务均等化水平还不高，改善民生任务仍然繁重；政府服务效能和社会管理水平有待提高。对此，我们要以对人民高度负责的态度，采取更加有力的措施认真研究解决。

　　 新一届政府目标任务

　　今后五年，是肇庆加快转型升级、实现跨越发展的关键时期。综观国内外形势，我市既面临重大发展机遇，又面临不少挑战，总体上机遇大于挑战。全球新一轮创新和产业升级日渐加快，绿色低碳经济和新兴产业发展方兴未艾；我国工业化、城镇化加快推进，经济发展长期向好趋势不变；我省加快转型升级，建设幸福广东，城市化和区域一体化发展提速推进；我市融入珠三角一体化发展步伐加快，发展后劲明显增强。与此同时，世界经济形势仍然严峻复杂，经济复苏不确定性、不稳定性上升，国家继续实施从紧的宏观政策，我市与珠三角核心区发展落差仍然较大。面对新起点、新机遇、新挑战，我们必须切实把握好中央经济工作会议提出的稳中求进的总基调，认清形势，把握机遇，应对挑战，奋发进取，扎实工作，努力开创科学发展新局面。

　　新一届政府工作总体要求是：高举中国特色社会主义伟大旗帜，以邓小平理论和“三个代表”重要思想为指导，深入贯彻落实科学发展观，按照市第十一次党代会决策部署，全面落实珠三角《规划纲要》和市“十二五”规划要求，加快转型升级步伐，大力实施“两区引领两化”战略，着力打造珠三角科学发展实验区、广东新型工业化基地、中国宜居文化名城、国际知名旅游会展胜地，加快建设经济发达、人民幸福、文明和谐、生态优美新肇庆，努力建设成为能够代表珠三角科学发展成果的城市。

　　新一届政府工作目标任务是：经济总量实现翻番，发展质量明显提高，城乡区域更加协调，社会事业全面进步，幸福指数不断提升。全市生产总值年均增长14%，人均生产总值年均增长13.3%，规模以上工业增加值年均增长20%，地方财政一般预算收入年均增长15%，万元生产总值能耗年均下降3.66%以上，城镇居民人均可支配收入年均增长10%，农村居民人均纯收入年均增长10%以上。珠三角《规划纲要》“四年大发展”和市“十二五”规划目标任务全面完成。

　　围绕上述总体要求和目标任务，必须着力推进以下重点工作：

　　一、以实施“两区引领两化”战略为重点，着力开创工业化和城市化发展新局面。全力推进肇庆高新区现代科技工业城建设，优化园区功能布局，重点发展先进制造业、高新技术产业、战略性新兴产业，构建现代产业体系，打造科技创新核心载体和高端创新人才集聚高地，依靠科技尽快实现产值五千亿，力争成为国家一流高新区。充分发挥肇庆高新区的示范辐射功能，大力实施增长引领、集群引领、科技引领和人才引领，促进全市产业布局优化、科技创新和人才聚集，加快全市新型工业化发展。全力推进肇庆新区建设，按照对接珠三角世界级城市群的要求和绿色低碳理念，高水平编制新区发展规划，以鼎湖区为主体，融合周边区域，规划建设行政服务、交通运输、商务会展、文化体育中心；科学选择主导产业，创新开发建设模式，力争“三年打基础、五年见成效、十年大跨越”，努力把肇庆新区建设成为科学发展的理想城市和城市化、信息化、工业化、市场化、国际化的重要载体。树立“绿色发展、智慧发展、包容发展、人本发展”的城市转型发展新理念，充分发挥肇庆新区的引领作用，统筹推进东南部城市群建设，提高山区城镇化水平，努力走出一条符合肇庆实际和可持续发展的新型城市化道路。

　　二、以加快转型升级为路径，着力建设现代产业新体系。坚持走转型发展、错位发展、低碳发展、幸福发展的道路，加快建设现代产业体系。积极调整优化产业结构，发展壮大第二产业，培育提升第三产业，继续优化第一产业，突出抓好先进制造业、高新技术产业、战略性新兴产业和生产性服务业，改造提升传统产业，促进优势产业集群化、新兴产业规模化、传统产业品牌化。积极谋划建设重大产业项目，实施主导产业招商，加快发展总部经济，推动骨干企业和主导产业集聚集约发展。推进自主创新，强化创新载体建设，完善主导产业和重点区域创新体系，积极引进培育创新人才团队、创新型企业和产学研创新联盟，提升产业核心竞争力。

　　三、以促进一体化为抓手，着力构建城乡区域协调发展新格局。落实珠三角“五个一体化”规划和广佛肇经济圈发展规划，逐步实现广佛肇基础设施共建共享、产业发展合作共赢、公共服务协调一体、生态环境协同保护。深化区域合作，协同推进粤桂产业合作示范区和广佛肇（怀集）经济合作区。实施肇庆主体功能区规划，形成主体功能清晰、发展导向明确、区域分工合理、经济人口资源环境协调的一体化发展格局。发挥中心区辐射带动功能。实施“双转移”，发展现代农业、特色工业和生态旅游，壮大县域经济规模实力。以水利、道路为重点统筹城乡基础设施建设，未来四年计划投入81亿元实施一批重点骨干水利项目，加大资金投入、加快农村公路改造建设。组织实施“十二五”规划建设的68项重点交通项目和29项重点能源项目，尽快实现“县县通高速”。

　　四、以发展社会民生事业为根本，着力创造幸福肇庆美好新生活。牢牢把握保障和改善民生这一根本目的，深入实施民生工程，切实办好为民惠民实事。坚持优先发展教育，大力推动县域城乡义务教育均衡发展，力争到2015年初步实现教育现代化。完善城乡一体化的公共就业服务体系和创业服务体系，争创省统筹城乡就业示范市。构建城乡一体化的社会保障体系，推进新型农村和城镇居民养老保险，发展慈善事业，完善社会救助体系和福利体系。建立健全以中低收入住房困难家庭为保障对象、以公租房为供给主体的新型住房保障制度，确保“十二五”期间城镇保障性住房覆盖面达到20%，完成农村低收入住房困难户住房改造任务。完善覆盖城乡居民的公共卫生和医疗卫生服务体系。加快文化强市建设，加强公共文化设施和文化服务体系建设，打造地方特色文化品牌，培育发展文化支柱产业，力争创建成为全国文明城市。加大生态环境保护力度，加强环保设施建设和环保监管，狠抓结构减排和环境综合整治，发展循环经济，确保完成污染减排目标。完善土地利用管理制度，提高节约集约用地水平。加快建设绿色休闲系统和生态景观林带。强化平安和谐肇庆建设，加强国防动员和双拥工作，维护社会公共安全。

　　五、以创新体制机制为突破，着力打造服务型政府新形象。牢牢把握加快改革创新这一强大动力，加大重点领域和关键环节改革力度。深化社会管理体制改革，加大政府职能转变管理力度，培育发展和规范管理社会组织。完善市场经济体制，推进财政管理体制改革、投融资体制改革、农村综合改革和市属国有企业体制改革。深化医疗保障制度改革，健全基本医疗保险筹资和待遇水平调整机制。深化行政审批制度和综合行政执法改革，推进县级大部制改革、事业单位人事制度改革和富县强镇、简政强镇事权改革。优化政府组织结构和运行机制，完善依法科学民主决策机制，着力建设法治服务政府。优化效能监察体系，完善惩治和预防腐败体系建设。

　　各位代表，站在发展新起点，面对人民新期盼，新一届市政府将牢记重托，不辱使命，瞄准新目标，抢抓新机遇，探索新路子，推动新发展，努力向全市人民交出一份满意的答卷！

　　 2012年工作安排

　　2012年是新一届政府工作的开局之年，我们将全面贯彻落实市第十一次党代会决策部署，紧紧围绕把肇庆建设成为能够代表珠三角科学发展成果城市这个核心，着力在加快转型升级上有新突破、在宜居城乡建设上有新面貌、在改善民生上有新成效，迈出科学发展、跨越发展的新步伐。

　　今年经济社会发展的主要预期目标是：生产总值增长13%，人均生产总值增长12%，一、二、三产业增加值分别增长4%、17.5%和11.6%以上，其中规模以上工业增加值增长20%；地方财政一般预算收入增长15%，固定资产投资增长20%，社会消费品零售总额增长18%，实际吸收外资和外贸进出口总额增长不低于全省增幅，城镇居民人均可支配收入增长10%，农村居民人均纯收入增长10.5%，居民消费价格涨幅控制在4.5%以内；人口出生率和自然增长率分别控制在省下达的计划指标以内，城镇登记失业率控制在2.45%以内，实现单位生产总值能耗和主要污染物减排目标。

　　实现上述目标，必须抓好以下十方面工作：

　　一、加快推进广佛肇经济圈建设。以经济圈建设促进转型升级，在做大经济总量的同时做优经济质量，确保全市经济平稳健康可持续发展。协调推进广佛肇重点合作项目和“十大工程”项目建设。推进广肇城际轨道建设，启动广佛肇高速公路工程，加快干线公路互通互达，促进三地交通网络“无缝对接”。深化与广佛产业合作，促进“双转移”实施，推进经济合作区建设。完善公共管理合作机制，推进环保、教育、医疗、卫生、综治、社保等全面合作对接。

　　二、加快推进新型工业化发展。抓好肇庆高新区扩园申报，加快推进中恒、国电等重大项目建设。加强园区科技创新体系建设，引进培育科技人才和创业团队。探索“一区多园”发展模式，推进各级园区、各类产业集聚基地建设，形成龙头引领、区域分工、园区集聚的发展格局。牢牢把握发展实体经济这一重点，继续培育壮大市工业50强，改造提升传统优势产业。抓好省市共建战略性新兴产业新型电子元器件基地建设。实施品牌战略，加大企业上市培育力度，拓宽企业融资渠道。落实扶持民营经济发展的政策措施，推动民营经济做大做强。严格环保准入，全面推行清洁生产，发展循环经济和低碳环保产业。

　　三、加快推进新型城市化建设。认真做好城市发展规划修编工作，抓紧完成肇庆新区规划，切实加强土地控规和土地储备，积极引进战略投资者，启动一批重点建设和基础设施项目，开展低碳城市建设试点。加快“三旧”改造步伐，创新城市管理体制，推进公共设施一体化，增强城市民生服务功能，大力改善城市人居环境。推进阅江大桥等一批市政重点工程建设，加快完成端州路过街隧道、西江路扩建改造等工程。认真做好迎接国家环境保护模范城市复检评审工作。提升旅游、金融、会展、房地产、文化创意、港口物流等规模和档次，建设特色街区和专业市场，加快培育市现代服务业30强。推动旅游景点景区和项目开发建设，在东南部谋划建设一批岭南特色小镇。加强节能减排和污染防治，开展星湖、羚山涌等环境综合整治。

　　四、加快推进招商引资和重大项目建设。坚持招商引资与择商选资相结合，突出“两区引领两化”主题，创新招商方式，力争引进一批投资超百亿的重大产业项目和具有核心竞争力的高端项目。加强重大项目谋划建设，加快二广高速怀集至连州段和至贺州段、江肇高速二期、肇花高速建设。积极配合贵广铁路、南广铁路、怀阳高速建设，抓紧筹划广肇高速扩建工程，启动市汽车客运总站搬迁建设。推进肇庆新港、三榕港扩建工程。继续抓好柳肇铁路、肇庆核电、中电国际热电联产等重大项目的前期工作。加快推进端州华南智慧城、城东“城市综合体”和鼎湖中储粮物流、广新星湖新材料等重大项目。

　　五、加快推进县域经济发展。加强中心镇和专业镇规划建设，努力打造全省城乡协调发展示范性山区县。科学规划建设产业集聚带，促进县域工业集聚集约发展。推动粤桂产业合作示范区建设，抓好德庆、怀集两个省级产业转移工业园规划调整申报，支持广宁华南再生资源工业基地申报省级产业转移工业园。充分发挥县域旅游资源和生态优势，发展特色旅游。完善农村消费市场和网点建设，努力扩大内需。推进四会精细化工基地、志高家电研发制造基地，高要大唐国际热电冷联产、华润新能源风电，广宁螺壳山风电、鼎丰公司增资扩产，德庆石井水泥三四期、盘龙湾旅游休闲，封开华润水泥、圆珠顶铜钼矿，怀集六祖禅院生态文化等县域大项目建设。

　　六、加快推进现代农业发展。全面落实强农惠农政策，加强“三农”建设，多渠道增加水利工程投入，建设农田水利万宗工程，加强大江大河整治和重点薄弱环节防洪除涝建设，推进星湖河湖联通、西江干堤加固等重点工程。发展现代农业，推广农业科技应用，扶持农业龙头企业和特色品牌，做强做优粮食、柑桔、蔬菜、禽畜、水产品、花卉六大主导产业，加强畜牧业标准化建设、疫情防控、禽畜品种改良。强化农产品质量安全监管，抓好省农产品质量安全检验检测体系建设项目。发展现代林业，扩大珍贵树种种植规模，改造鼎湖山周边纯松林生态，推进现代林业交易平台体系建设。加快生态文明村和名村示范村建设。

　　七、加快推进体制改革创新。着手开展医疗保障体制、住房保障制度、培育发展和规范社会组织三大重点改革。推进行政体制机制改革，健全覆盖政府性资金和财政运行全过程的财政监督机制，完善市对端州区、高新区财政管理体制。推进市属国有资产统一归口管理，支持国有企业做强做优。推进农村综合改革，加快农村土地承包经营权流转，发展政策性农业保险。完善科技创新服务体系，充分发挥肇庆高新区、产学研结合示范基地等创新载体对高新技术产业和战略性新兴产业的集聚作用，强化孵化器建设。支持骨干企业组建国家级、省级重点实验室、工程技术研发中心等创新平台，争取10家以上企业认定为高新技术企业，吸纳更多科技特派员进驻企业，全面提升科技创新水平。

　　八、加快推进社会民生事业发展。积极争创全国文明城市。大力发展文化旅游产业，培育发展新兴文化，打造一批国家级、省市级文化产业示范基地和景区景点。完成市图书馆新馆建设，筹建市博物馆新馆、市档案馆、市文化馆、市青少年宫。实施肇庆古宋城保护与开发项目，抓好崇禧塔、阅江楼、梅庵等文化景区扩建整治修复，加强文化遗产保护。完成第三次文物普查。加强文化市场管理。扶持文艺精品创作。深入推进教育强市创建工作，确保德庆、封开创建为省教育强县，确保年底前通过创建省教育强市验收。加快建设优质幼儿园、中小学校，支持肇庆学院等高等院校提高办学水平和层次，推动校企联合培养技能人才，推进肇港澳职业教育合作。完成市第一人民医院新院、市中医院新住院大楼建设，推进市第三人民医院新住院楼等项目建设，新增10个以上省卫生镇。开展农村劳动力技能培训和转移就业，统筹推进高校毕业生、农民工和就业困难人员就业。做好农民工“积分制入户”工作，推进劳动关系和谐企业与工业园区“双创建”活动。完善社会保障体系，推进“金保工程”建设，提高城镇和农村低保标准，把山区县农村家庭年人均收入低于2500元的困难群众纳入低保范围，对80岁以上老人发放高龄津贴。做好人口计生工作，稳定低生育水平。加强国防动员和双拥工作，力争创建成为省双拥模范城。积极申办第十五届省运会，推动全民健身。继续抓好统计、外事、侨务、港澳、民族、宗教、老区建设、人防、地震、气象、档案、地方志、妇女儿童、老龄、残疾人、慈善等各项工作。

　　九、加快推进社会管理和创新。建立完善社会工作机构，规范社区公共服务站建设，培育发展社区社会组织。健全防灾减灾体系，创建100个全国综合减灾示范社区。加强食品、药品、产品质量安全监督，严厉打击制假售假、走私贩私、非法传销。落实安全生产“一岗双责”制度，加强安全生产监管，健全突发事件应急处置机制和110社会抢险救援联动机制，保障群众生命安全。加强价格巡查监控，维护市场价格稳定。强化综治信访维稳三级平台建设，健全社会治安立体化防控体系，完善城区和重点镇街治安视频监控设施，加强排查和预警，及时掌握影响社会安全稳定的隐患，妥善处理各种矛盾纠纷，确保社会大局和谐稳定。

　　十、加快推进服务型政府建设。自觉接受人大及其常委会依法监督和人民政协民主监督，严格执行各项决议决定，定期向市人大及其常委会报告工作和向市政协通报情况，办好人大代表建议和政协委员提案，认真听取民主党派、工商联、无党派人士、各人民团体和专家顾问意见建议。加强依法行政，创建民主法治示范村（社区）。完善科学发展观绩效考核评价制度，创新公务员管理监督和激励机制。推进服务型政府建设，继续开展机关作风评议和“人民群众满意服务窗口”创建活动，办好“行风热线”和网络问政平台。完善网上联合审批系统，提升政府部门在线办公水平。加强专项治理和监督检查，切实解决企业和人民群众反映强烈的热点、难点问题。推进政府廉政建设，加强行政监察和审计监督，规范权力运行。

　　各位代表，多为群众办好事、办实事是政府工作的出发点和落脚点，市政府今年将继续集中力量办好十件惠民实事：一是加快公租房建设。建立健全以公共租赁住房为主的保障性住房制度，新增保障性住房3182套（其中市城区1280套），以解决中低收入家庭、新就业无房人员和在城镇稳定就业的外来务工人员住房困难。二是完成困难户住房改造。全面完成全市9693户农村低收入住房困难户住房改造建设任务，让困难群众住有所居。同时按省标准全面完成扶贫开发任务。三是不断扩大社会就业。全市新增就业89000人，其中城镇新增就业45400人，农村培训转移就业40000人，大学生就业3600人。四是实施价格惠民工程。全市新建平价商店30家。对按政府要求承担平抑价格责任的平价商店给予政策扶持，对因价格上涨而影响基本生活的低收入群众给予临时价格补贴。五是为困难学生发放补贴。对全市2.8万名农村中小学义务教育阶段家庭经济困难学生给予每人每年600元膳食和交通补贴。六是开展“文化三下乡”。全市送千台演出、万场电影、百万册图书下农村进基层，让广大人民群众共享幸福肇庆的文化成果。七是加快城乡道路改造。完成农村公路路面硬底化建设里程300公里，端州和鼎湖城区分别各改造10条和5条以上旧街旧巷，切实改善群众出行条件。八是启动星湖水质整治。加强星湖科学规划和保护，加快实施星湖截污和引水工程，切实改善星湖水质，净化、美化星湖环境。九是加快绿色肇庆建设。建设生态景观林带2.1万亩，新增投放100台新能源公交车，推进节能减排，打造绿色生态屏障，营造优美的生态环境。十是启动“幸福社区”工程。以“六个好”为标准，创建38个幸福社区，创新社区管理服务，改善社区生活环境，不断提升人民群众的幸福感。

　　各位代表！肇庆正站在新的历史起点上，新形势新任务催人奋进，新的使命艰巨而光荣。让我们在中共肇庆市委的正确领导下，深入贯彻落实科学发展观，紧紧依靠全市人民，把握机遇，开拓进取，同心协力，为把肇庆建设成为能够代表珠三角科学发展成果城市而努力奋斗！
